# Methotrexate polyglutamates as a potential marker of adherence to long-term therapy in children with juvenile idiopathic arthritis and juvenile dermatomyositis: an observational, cross-sectional study

**DOI:** 10.1186/s13075-015-0814-z

**Published:** 2015-10-22

**Authors:** Ahmed F. Hawwa, AbdelQader AlBawab, Madeleine Rooney, Lucy R. Wedderburn, Michael W. Beresford, James C. McElnay

**Affiliations:** Clinical and Practice Research Group, School of Pharmacy, Queen’s University Belfast, 97 Lisburn Road, BT9 7BL Belfast, UK; Aston Pharmacy School, Aston University, Birmingham, UK; Faculty of Pharmacy, Al Zaytoonah University, Amman, Jordan; Centre for Infection and Immunity, School of Medicine and Biomedical Sciences, Queen’s University Belfast, Belfast, UK; Institute of Child Health, University College London, London, UK; Arthritis Research UK Centre for Adolescent Rheumatology, University College London, University College London Hospital, London, UK; Department of Rheumatology, Great Ormond Street Hospital NHS Foundation Trust, London, UK; Department of Women’s and Children’s Health, Institute of Translational Medicine, University of Liverpool, The Alder Hey Children’s NHS Foundation Trust, Liverpool, UK

## Abstract

**Introduction:**

Methotrexate (MTX) is a cornerstone of treatment in a wide variety of inflammatory conditions, including juvenile idiopathic arthritis (JIA) and juvenile dermatomyositis (JDM). However, owing to its narrow therapeutic index and the considerable interpatient variability in clinical response, monitoring of adherence to MTX is important. The present study demonstrates the feasibility of using methotrexate polyglutamates (MTXPGs) as a biomarker to measure adherence to MTX treatment in children with JIA and JDM.

**Methods:**

Data were collected prospectively from a cohort of 48 children (median age 11.5 years) who received oral or subcutaneous (SC) MTX therapy for JIA or JDM. Dried blood spot samples were obtained from children by finger pick at the clinic or via self- or parent-led sampling at home, and they were analysed to determine the variability in MTXPG concentrations and assess adherence to MTX therapy.

**Results:**

Wide fluctuations in MTXPG total concentrations (>2.0-fold variations) were found in 17 patients receiving stable weekly doses of MTX, which is indicative of nonadherence or partial adherence to MTX therapy. Age (*P* = 0.026) and route of administration (*P* = 0.005) were the most important predictors of nonadherence to MTX treatment. In addition, the study showed that MTX dose and route of administration were significantly associated with variations in the distribution of MTXPG subtypes. Higher doses and SC administration of MTX produced higher levels of total MTXPGs and selective accumulation of longer-chain MTXPGs (*P* < 0.001 and *P* < 0.0001, respectively).

**Conclusions:**

Nonadherence to MTX therapy is a significant problem in children with JIA and JDM. The present study suggests that patients with inadequate adherence and/or intolerance to oral MTX may benefit from SC administration of the drug. The clinical utility of MTXPG levels to monitor and optimise adherence to MTX in children has been demonstrated.

**Trial registration:**

ISRCTN Registry identifier: ISRCTN93945409. Registered 2 December 2011.

## Introduction

Methotrexate (MTX) is an effective disease-modifying antirheumatic drug (DMARD) widely used in children to treat a variety of autoimmune and inflammatory conditions, including juvenile idiopathic arthritis (JIA) and juvenile dermatomyositis (JDM) [1–6]. Owing to its efficacy and cost-effectiveness, MTX is likely to remain a cornerstone of treatment [5, 7]. However, because of the considerable interpatient variability in clinical response, absorption difficulties and a wide spectrum of side effects, monitoring of adherence to MTX is important [4, 8–11].

MTX is a prodrug activated intracellularly to form methotrexate polyglutamates (MTXPGs) through sequential addition of glutamic acid residues by the enzyme folylpolyglutamate synthase (FPGS) [12, 13]. Within the cell, MTXPGs bind to and inhibit dihydrofolate reductase and other folate pathway enzymes required for purine and pyrimidine synthesis, thereby providing anti-inflammatory effects [14, 15]. Polyglutamation of MTX promotes its retention intracellularly, resulting in enhanced inhibitory effects against its target enzymes. Compared with MTX itself, long-chain MTXPGs have been shown to confer much more potent inhibition of target enzymes within folate metabolism [13, 16, 17]. The clinical value of monitoring serum concentrations of the parent drug is therefore very limited and generally not practical, because approximately 95 % of MTX dose is metabolised within 24 h of administration [13, 18, 19]. However, monitoring MTXPGs as a surrogate biomarker for drug exposure offers a potential tool to assess adherence to long-term therapy in patients with chronic inflammatory conditions (due to their long half-life of elimination) [20, 21]. Evidence suggests that MTXPGs may be associated with the efficacy and toxicity of MTX treatment in rheumatoid arthritis, and some investigators have advocated routine monitoring of MTXPGs for that reason [22–25].

Apart from one recent study in children with JIA [26], adherence rates to MTX treatment in paediatric patients with rheumatologic diseases, including JIA and JDM, have not been previously reported. Studies investigating MTX adherence in adult rheumatology patients have been based mainly on prescription data [27–29], electronic pill counts [30] or self-reported measures [31]. Reported nonadherence to medications in adult patients with JIA ranges from 8 to 48 %, with rates fluctuating depending on the time of evaluation and method used for adherence assessment [32–37]. Risk factors for lack of adherence could include demographic variables such as the patient’s being an adolescent or having low socioeconomic status [32, 34, 38], having concerns about side effects of administered drug or lack of belief about the necessity of treatment [31, 39].

In the present study, adherence to MTX treatment in a cohort of children with JIA and JDM was evaluated. A method for measuring MTXPG concentrations using the novel dried blood spot (DBS) sampling technique was developed by our group [40] and applied in the present study to evaluate adherence to prescribed MTX therapy in children. A secondary aim of the study was to characterise the pattern of variability in MTXPG concentrations and determine the clinical variables and biochemical parameters that could predict the observed variability in MTX metabolite levels.

## Methods

### Study population

A total of 49 paediatric patients with JIA or JDM were recruited into this study from three paediatric outpatient rheumatology clinics in three different centres: (1) Musgrave Park Hospital, Belfast, Northern Ireland; (2) Alder Hey Children’s NHS Foundation Trust, Liverpool, UK; and (3) Great Ormond Street Hospital for Children NHS Foundation Trust, London. Patients between the ages of 4 and 17 years who were receiving stable MTX weekly doses [oral or subcutaneous (SC)] for at least 2 months were identified for inclusion in the study.

Participants were asked to provide one DBS sample for analysis of MTX metabolite content during a regularly scheduled clinic visit. Samples were collected by finger prick from each child by spotting drops of blood (two spots per child) directly onto a Guthrie card. For children 5 years of age or older, parents and/or older children were also asked to take two additional DBS samples at home 4 weeks apart, dry them overnight at room temperature and mail them to our laboratory in prepaid mailer kits for analysis.

Accurate information about MTX dosing and times of sampling were recorded prospectively during the clinic visit using bespoke collection forms. The following data were also collected from patient case notes and clinical chemistry records and recorded in these collection forms: age, weight, height, medical history (date of diagnosis, other medical conditions and current medications), biochemical parameters, clinical outcome and records of side effects experienced (e.g., nausea and vomiting). For samples obtained at home, the mailer kits included appropriate collection forms mailed to the patient and/or the patient’s parent in advance of the sampling time, with clear instructions on how to take the sample and fill in the form.

### Ethical considerations

The study was approved by the Office for Research Ethics Committees in Northern Ireland (reference number 10/NIR03/33). Patients were included in the study only after their parents or legal guardians had been fully informed and had signed the study consent form. In addition, verbal or written assent was obtained from older children (≥6 years) before enrolment into the study.

### Measurement of methotrexate polyglutamate concentrations

DBS concentrations of MTX active metabolites (MTXPGs up to the fifth order of glutamation, MTXPG_5_) were measured by using a selective and sensitive reverse-phase liquid chromatography–tandem mass spectrometry (LC-MS/MS) methodology that we previously developed and validated [40]. Intraday and interday coefficients of variation were less than 15 %, and the limits of detection for individual MTXPGs (MTXPG_1–5_) and total concentration of methotrexate polyglutamate (MTXPG_total_) were determined at 1.6 nmol/L and 1.5 nmol/L, respectively.

Differences in MTXPG concentrations between patients were evaluated as a function of age, sex, diagnosis and route of administration (SC or oral). To differentiate the effect of these factors from dose- or weight-related changes, MTXPG concentrations were normalised by administered dose of MTX (per kilogram of patient weight or per square metre of body surface area) and expressed as nanomoles per litre per milligram or nanomoles per litre per milligram per square metre.

### Assessment of adherence

The pattern of variability in MTXPG levels as a function of time was evaluated over two consecutive 4-week intervals during which patients were receiving stable MTX doses. Nonadherence was assumed if a patient had wide variations (≥2.0-fold) in MTXPG levels on different occasions while prescribed the same dose of MTX. A ratio of 2.0-fold was chosen as the cutoff point because similar values have been suggested for identifying patients nonadherent to long-term therapy in chronic diseases [41, 42].

### Statistical analysis

Statistical analysis was performed using IBM SPSS version 21 software (IBM SPSS, Armonk, NY, USA). The results were expressed as the median and range values or as frequencies. Descriptive statistics were used to characterise the variability in individual MTXPG_1–5_ and MTXPG_total_ concentrations between different groups of patients. Univariate associations between MTXPG concentrations and demographic or clinical characteristics were analysed using the Mann–Whitney *U* test or the Kruskal–Wallis test, as appropriate. Group differences in the proportion of patients with ≥2.0-fold variation in their MTXPG levels were explored using χ^2^ tests with 1 degree of freedom or Fisher’s exact test, as appropriate. Comparison of the median fold ratio of highest to lowest MTXPG concentration was performed using the Mann–Whitney *U* test. All analyses were two-sided, with *P* values <0.05 considered significant.

## Results

### Demographic characteristics of the study population

Of the 49 patients recruited into the study, 32 (65 %) had an underlying diagnosis of JIA and 17 had JDM. The median age (range) of the population was 11.5 years (4–17 years), and 67 % were female. All patients were being treated with weekly MTX (median 0.31 mg/kg, equivalent to 10.2 mg/m^2^) for long durations (median 2.8 years, 5 months–10 years), and 41 % were receiving SC MTX. One patient did not provide a DBS sample at the clinic and therefore was not included in the analysis. Of the patients included in the analysis (*n* = 48), 40 (83 %) provided at least two DBS samples during the study period and 60 % received oral folic acid supplementation (median dose 5 mg/week). The demographic and clinical characteristics of the study participants are shown in Table 1.

**Table 1:** Demographic and clinical characteristics of study patients

Characteristics	Values^a^ (*n* = 49)
Age (yr)	11.5 (4–17)
Sex [females, n (%)]	33 (67.3 %)
Weight (kg)	41 (13.4–119.7)
Height (cm)	146 (95–178)
MTX weekly dose (mg)	13.75 (7.5–20)
MTX weekly dose (mg/kg)	0.31 (0.08–0.67)
MTX weekly dose (mg/m^2^)^b^	10.2 (4.1–15.5)
Number of medications prescribed	5 (1–8)
Number of side effects recorded	3 (1–2)
Pain scale^c^	0.9 (0–7.1)
General evaluation scale^c^	0.5 (0–8.2)
CMAS score^d^	51.5 (42–55)

^a^Values represent median (range), except where indicated otherwise

^b^Surface area was calculated using the Mosteller method [59]

^c^Pain scale and general evaluation scores were obtained from the patients’ Childhood Health Assessment Questionnaire

^d^Childhood Myositis Assessment Scale (CMAS) score was recorded for children with JDM

### Distribution of methotrexate polyglutamate concentrations and effect of administered dose

In our cohort of 48 patients with clinic DBS samples, the median MTXPG_total_ was 91.5 nmol/L (range 3.5–470.4 nmol/L). Analysis of the correlation between MTXPG_total_ and individual MTXPG subtypes revealed a very high correlation (*R* = 0.91, *P* < 0.0001) between total MTXPGs and MTX triglutamate (MTXPG_3_). Considerable internal correlations of the concentrations within each MTXPG subgroup were also observed; that is, short-chain MTXPGs MTXPG_1–2_ were highly correlated with each other (*R* = 0.73, *P* < 0.0001), as were very long chain polyglutamates MTXPG_4–5_ (*R* = 0.61, *P* < 0.0001).

**Fig. 1:**
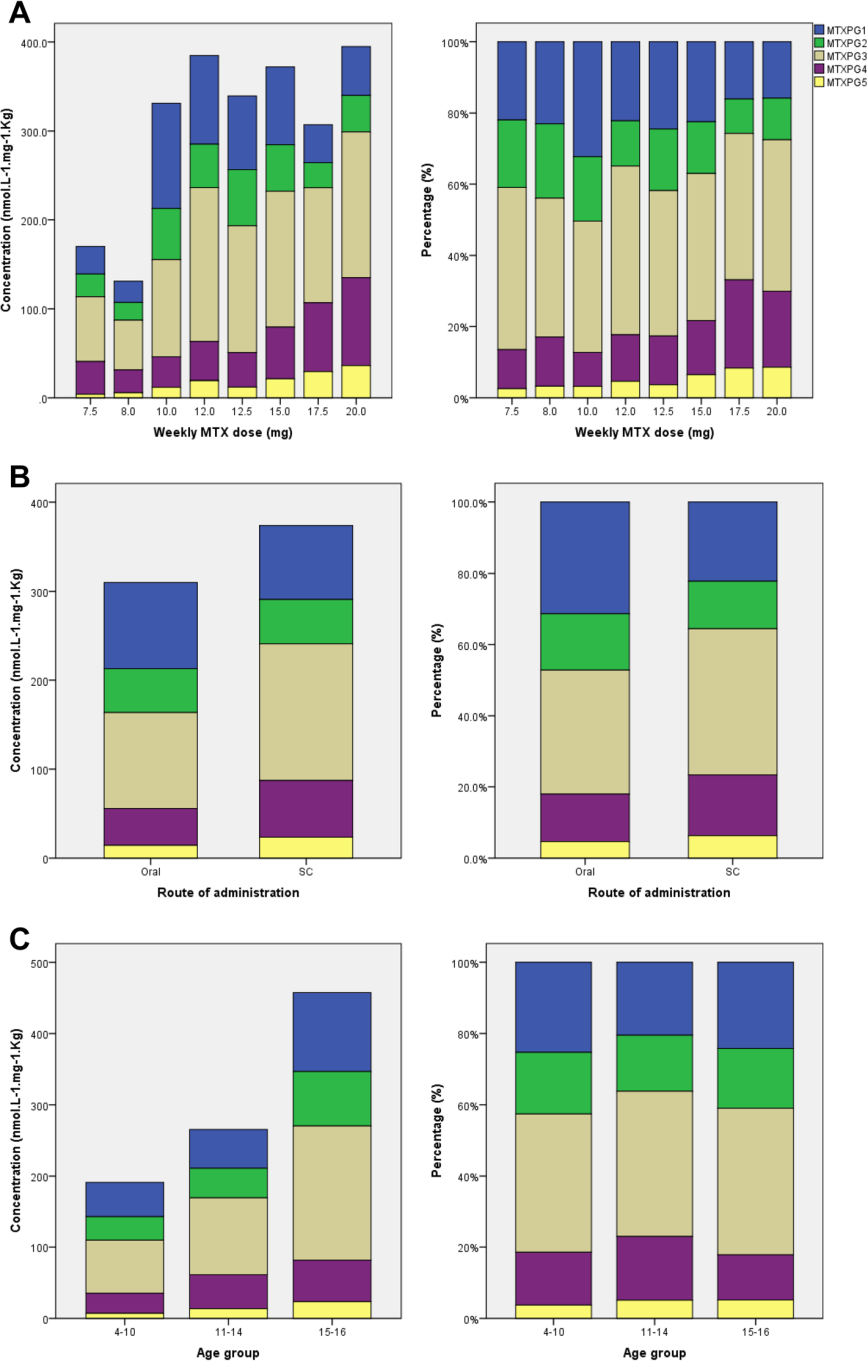
Effect of administered dose (**a**), route of administration (**b**) and patient age (**c**) on the distribution of methotrexate (MTX) polyglutamates in children receiving long-term MTX therapy

The median concentration of MTXPG_total_ was significantly greater when higher MTX doses were administered (*R* = 0.27, *P* = 0.0007). This appears to be attributable to selective accumulation of long-chain MTXPG_3_ (*R* = 0.28, *P* = 0.0005) and very long chain MTXPG_4–5_ (*R* = 0.39–0.42, *P* < 0.0001) at the expense of short-chain MTXPG_1–2_. This was confirmed by the percentage change in individual MTXPGs (relative to MTXPG_total_) as a function of MTX dose (Fig. 1a). Interestingly, concurrent administration of higher folic acid doses was associated with increased concentrations of shorter-chain MTXPG_1–2_ (*R* = 0.36–0.42, *P* = 0.002). Similarly to previous reports [43], the dose of MTX was higher in patients taking folic acid supplementation.

### Association of methotrexate polyglutamate levels with clinical variables

Bivariate analysis revealed significantly higher MTXPG_total_ concentrations in patients who had MTX administered by the SC route than among patients who received the drug orally (median 114.1 nmol/L after SC administration vs. 75.5 nmol/L after oral administration; *P* < 0.0001). When corrected for the dose administered (i.e., relative to 1 mg/kg or 1 mg/m^2^ weekly doses of MTX), the difference in MTXPG_total_ concentrations stratified by route of administration remained significant (median concentration 307.8 nmol/L/mg/kg vs. 236.2 nmol/L/mg/kg dose after SC and oral administration, respectively; *P* = 0.004; such concentrations are equivalent to 11.0 nmol/L/mg/m^2^ vs. 7.41 nmol/L/mg/m^2^, respectively; *P* = 0.002).

Furthermore, marked differences in the distribution of individual MTXPG concentrations according to the route of administration were observed. Both normal and dose-adjusted concentrations of long-chain MTXPG_3_ and longer-chain MTXPG_4–5_ were higher in patients who received SC MTX than in patients receiving MTX. Evaluation of the proportions of individual MTXPG subtypes (relative to MTXPG_total_) also supported these findings. The median proportions of long-chain MTXPG_3_ and MTXPG_4_ were higher in patients treated subcutaneously (*P* = 0.002 and *P* < 0.0001 for MTXPG_3_ and MTXPG_4_, respectively), whereas the short-chain MTXPG_1–2_ made up larger proportions of MTXPGs in patients treated orally (*P* = 0.001 and *P* < 0.0001 for MTXPG_1_ and MTXPG_2_, respectively) (Fig. 1b).

MTXPG_total_ concentrations did not change significantly with age (*P* > 0.05), despite the fact that older children received significantly lower doses (per kilogram and per square metre) of MTX (*P* < 0.0001). When corrected for dose administered, however, both MTXPG_total_ and individual MTXPGs were significantly higher in older children receiving MTX (*P* < 0.0001 for MTXPG_total_; *P* < 0.01 for MTXPG_1–2_; and *P* < 0.001 for MTXPG_3–5_) (Fig. 1c). These findings are indicative of lower clearance rates of MTXPGs in older children than in younger children.

Whether the patient had an underlying diagnosis of JIA or JDM was significantly associated with varying proportions of individual MTXPG subtypes. In particular, patients who had a diagnosis of JIA had significantly greater proportions of short-chain MTXPG_1–2_ (*P* < 0.005), whereas long-chain MTXPG_3_ and very long-chain MTXPG_4_ made up larger proportions of total MTXPGs in patients diagnosed with JDM (*P* < 0.001 and *P* = 0.009, respectively). Such differences could reflect the fact that higher numbers of patients were treated orally in the JIA subgroup (64 % treated orally in the JIA subgroup vs. 41 % in the JDM subgroup).

### Association of methotrexate polyglutamate levels with biochemical parameters

Increased dose-normalised MTXPG_total_ levels were associated with greater antiarthritic effect and improved likelihood of disease control, as suggested by reduced levels of the inflammation markers C-reactive protein (*P* = 0.026) and erythrocyte sedimentation rate (ESR; *P* = 0.033), in patients who had higher accumulation of MTXPG_total_ concentrations.

### Assessment of adherence to methotrexate therapy

**Fig. 2:**
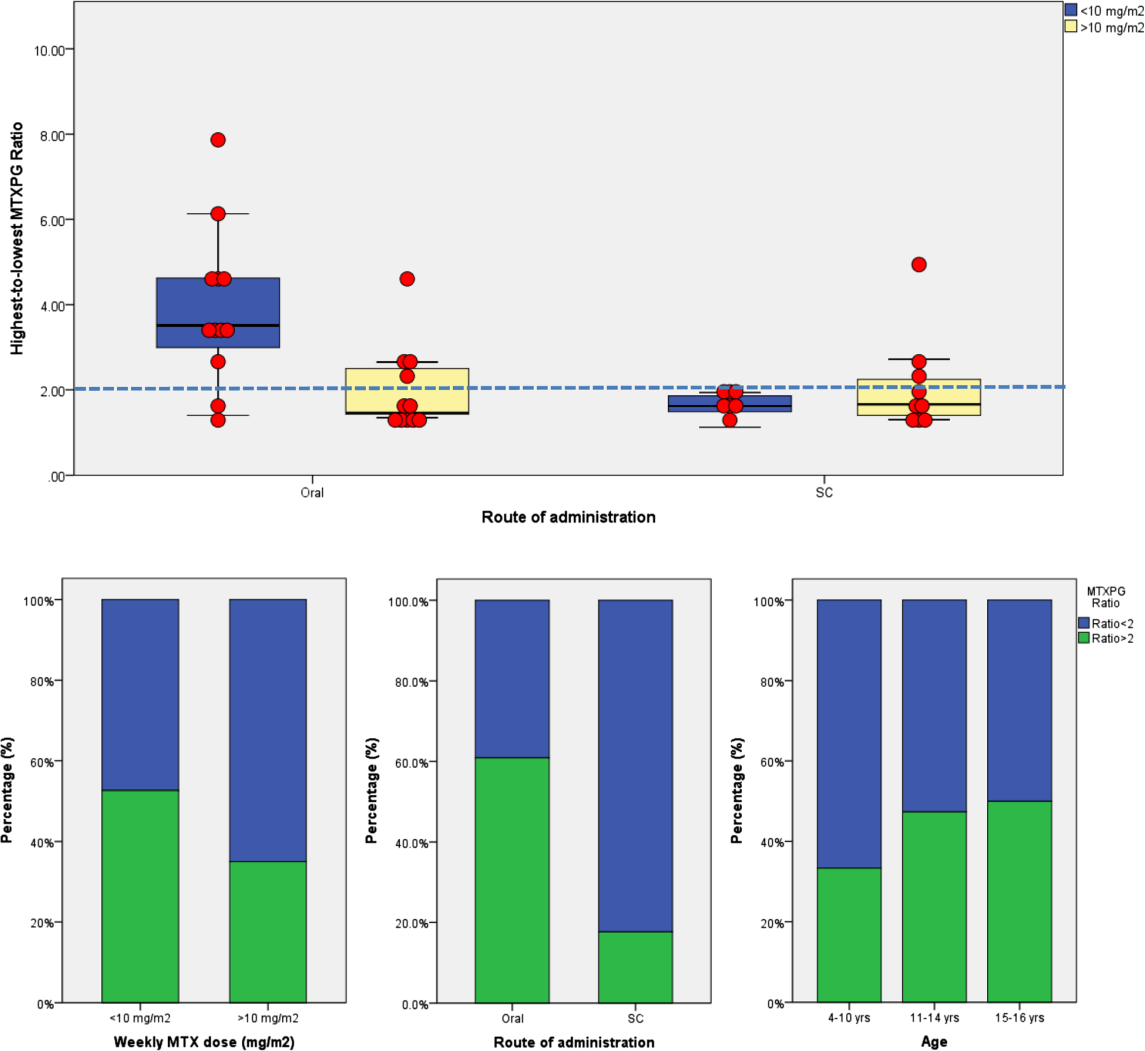
Comparison of the effects of weekly methotrexate (MTX) dose, route of administration and patient age on the highest to lowest methotrexate polyglutamate (MTXPG) ratio recorded in children receiving long-term MTX therapy. *Dashed reference line* represents a ratio >2.0-fold, which is indicative of wide fluctuations in MTXPG concentration

Highest and lowest MTXPG levels were recorded for each patient who had at least two measurements of their metabolite levels during the study period (*n* = 40). Nonadherence or partial adherence to MTX therapy was identified in 17 (42.5 %) of the patients, in whom greater than 2.0-fold variations in MTXPG_total_ concentrations were observed. Of these 17 patients, 5 (29 %) were male and 12 (71 %) were female. They ranged in age from 7 to 17 years (median 14 years). At the time the samples were taken, these children were supposed to be taking between 10 and 20 mg weekly MTX doses without change for at least 5 months. Of note, highest to lowest MTXPG ratios, indicative of nonadherence, were significantly greater in patients receiving smaller doses (measured as milligrams per square metre) (*P* < 0.031) (Fig. 2). This suggests that patients with lower disease activity (in whom doses were not increased as they grew) could be associated with an increased risk of poor adherence to therapy. This was particularly apparent when patients were given MTX orally in the present study (Fig. 2). Overall, the results suggest that nearly half of children may fail to fully comply with MTX doses as prescribed during their long-term therapy.

### Effect of route of administration and age of child on adherence to methotrexate

Univariate analyses showed that both the route of administration and patient age were significantly associated with nonadherence to MTX treatment (Fig. 2). A higher proportion of patients who received SC MTX were found to be adherent to their medication. Only 17.6 % had greater than 2.0-fold variation in their MTXPG levels after SC administration versus 60.8 % of patients who received MTX orally (*P* = 0.005). Furthermore, adolescents and older children were more likely than younger children to be nonadherent to their prescribed medication; older age was significantly correlated with greater MTXPG ratio (*R* = 0.35, *P* = 0.026).

Sex as a variable was similarly distributed between adherent and nonadherent patients. In addition, there were no statistically significant differences in the underlying diagnosis (JIA vs. JDM) or other obvious sociodemographic characteristics between the nonadherent group and the remainder of the cohort (*P* > 0.05).

### Association of nonadherence with biochemical parameters and gastrointestinal toxicity

The frequency of MTX-related gastrointestinal (GI) side effects (particularly the presence of nausea or vomiting) in nonadherent patients compared with the rest of the cohort were evaluated only in a subset of patients, because reports of nausea and vomiting were recorded only prospectively in 19 patients of those enrolled in the study. Of these 19 patients, 9 reported nausea, vomiting or other GI side effects at some stage during the study. Seven (78 %) of these patients had greater than 2.0-fold variation in their MTXPG levels and therefore were considered nonadherent to therapy. The mean MTXPG ratio in patients who reported nausea or vomiting was 3.5-fold compared with 2.4-fold in those who did not have GI complaints. These findings suggest that the presence of MTX-related side effects could increase the risk of poor adherence to MTX in children with JIA and JDM. It is also possible that the nonadherent group did not follow folic acid supplementation prescribed to reduce MTX toxicity.

Finally, examining the differences in biochemical parameters between the adherent and nonadherent groups showed a significant association between nonadherence and elevated ESR levels (*P* < 0.043), suggesting an increased level of inflammation and poor disease control in nonadherent patients. This association was higher in patients receiving MTX orally (*P* = 0.035).

## Discussion

To our knowledge, this is the first study in which the extent of MTX adherence in paediatric patients with JIA and JDM has been evaluated on the basis of DBS measurement of MTXPGs. The approach using DBS proved an acceptable alternative to the use of larger whole blood or red blood cell sample quantities and had the advantage of being minimally invasive, allowing parents and older children to take blood samples at home. The combination of sparse sampling and low sample volume helped to overcome ethical and practical difficulties associated with traditional blood sampling in children and was shown to be useful in estimating adherence.

Similarly to previous reports [44, 45], the present study demonstrates significant interpatient variability in total and individual MTXPG concentrations. Such variability was explained, at least in part, by MTX administered dose, route of delivery and patient age. Higher MTX doses were associated with greater percentage of longer-chain MTXPG_3–5_ at the expense of short-chain MTXPG_1–2_. Such selective enrichment of longer-chain MTXPGs at higher doses is consistent with recent observations in children with JIA and children with acute lymphoblastic leukaemia [45–47]. Similarly, MTXPG distribution differed by the route of administration: Patients receiving SC MTX had higher overall levels of MTXPGs as well as selective accumulation of longer-chain MTXPGs. This route-dependent distribution of MTXPGs persisted when concentrations were corrected relative to administered dose (measured as milligrams per kilogram or milligrams per square metre). A plausible explanation for such route-specific and dose-dependent accumulation of long-chain MTXPGs could be the higher concentrations produced following SC delivery or higher-dose administration of MTX. It has been suggested that higher concentrations can result in a more pronounced folate-depleted state in cells, which activates a feedback mechanism that upregulates polyglutamation [45]. The stability of longer-chain polyglutamates would in turn allow for the preferential retention and accumulation of long-chain MTXP_3–5_.

Additional factors likely to be significant contributors to MTXPG variability include pharmacokinetic variation, physiological and developmental differences in oral absorption, transporter expression and the individual genotypes for MTX multiple metabolic pathways that have been associated with both efficacy and toxicity of MTX [48–50]. Because of the importance of folate in growth and development in children, concurrent administration of folic acid supplementation might also explain some of the differences in MTXPG concentrations. This is of particular importance in older children with higher growth demands in puberty.

Folic acid supplementation is routinely given to reduce potential side effects in patients receiving MTX [51, 52]. However, because MTX and folate are transported by the same transporter within cells and compete for FPGS for polyglutamation, it is suggested that higher intracellular folate levels may trump MTX retention and affect its polyglutamation [53, 54]. The present study shows that folic acid use in MTX-treated children was associated with increased concentrations of short-chain MTXPG_1–2_ at the expense of longer-chain MTXPG_4–5_. Such an impact on MTX biotransformation leads to either reduced MTX efficacy or higher MTX dose requirements to achieve the same clinical effect in the absence of folic acid.

Although inherited differences in metabolism may explain interpatient variability, they should not cause intrapatient variability once a steady state is reached. Such variability is hard to explain on any grounds other than not taking the medication as prescribed. Other factors, such as diet and alcohol intake, in teenagers could aggravate this behaviour of nonadherence to prescribed medicines. An approach to detect nonadherence in the present study was therefore to study patients at different times while they were prescribed the same dose of MTX by the same route. If there were a wide variation in MTXPG_total_ concentrations at steady state, this would most likely be a reflection of poor adherence.

According to this method, nearly half the patients had wide fluctuations in their MTXPG concentrations indicative of partial adherence or nonadherence. Interestingly, doses received by nonadherent children in the present study were significantly lower (measured as milligrams per square metre) than those prescribed to children who were assumed to be adherent to MTX therapy. This increased variability can be explained partly by the children dosed with the lowest oral doses, because such doses will have a greater proportion of short-chain MTXPGs that are susceptible to more significant and rapid concentration shifts. It could also indicate that children who received lower doses did not perceive a high necessity for MTX therapy, owing to their low disease activity, and therefore were less likely to take their medication consistently. Conversely, lower doses could indicate an attempt to improve adherence in patients who were more prone to MTX side effects. Further studies are required, however, to confirm these hypotheses.

Other risk factors for nonadherence to MTX therapy include GI side effects associated with the administration of MTX. Seven of the nine patients who reported nausea and vomiting at some stage during the present study had greater than 2.0-fold variation in their MTXPG levels and therefore were considered nonadherent to therapy. Such side effects can be diminished with SC administration of MTX. Patients receiving the drug subcutaneously are therefore expected to show better adherence than those treated orally [55]. Indeed, compared with patients who received the drug orally, a higher proportion of patients who received SC MTX in the present study were found to be adherent to their medication. A possible explanation for better adherence with the SC mode of administration could be the greater antiarthritic effect and better clinical outcome among patients following SC MTX administration compared with those taking oral MTX, as reported in previous studies [55–57]. However, an obvious difference impacting adherence is the fact that SC administration is more likely to be provided by a caregiver and thus is more likely to be observed.

In the present study, adherence did not relate to sex or disease type. However, a significant association between older age of children and nonadherence was established. This is concordant with previous studies that recognised adolescents as having lower adherence than younger children because of their growing autonomy [32, 34, 58]. Older children and adolescents, therefore, should be monitored carefully, particularly when they experience less severe symptoms during the stable phases of their disease. In general, adherent patients had better disease control throughout the study. This was supported by elevated ESR levels, a marker of inflammation, in nonadherent patients.

A precise, sensitive and selective LC-MS/MS assay was used for determination of MTXPGs in the present study. Although LC-MS/MS-based methods are increasingly used in clinical laboratories, the cost involved in sample preparation and access to the instrument might limit its use for routine monitoring of adherence in the clinical setting. Another limitation of the current assay is inability to measure the intracellular levels of relevant folate species. The novel DBS sampling, on the other hand, has the advantage of being both convenient for home sampling and a direct objective measure of adherence. An additional obvious benefit is the lower blood volume requirement applicable for monitoring MTXPGs in children.

## Conclusions

The present study is unique in being the first detailed analysis of MTXPG levels based on DBS measurements in JIA and JDM. Our data highlight the significance of nonadherence to MTX therapy in patients with JIA and JDM and demonstrate the feasibility of measuring MTXPGs in DBS samples as a potential tool to monitor adherence. We also explored the pattern of variability in MTXPG concentrations and its association with clinical variables and additional biochemical parameters pertaining to disease activity and MTX side effects. The findings warrant further investigation into the clinical utility of MTXPG levels to guide therapy and optimise adherence to MTX treatment in children. Furthermore, the study highlights the importance of understanding the potential barriers to MTX adherence in order to help manage the disease and improve patients’ quality of life.

## Abbreviations

CMAS: Childhood Myositis Assessment Scale
DBS: Dried blood spot
DMARD: Disease-modifying antirheumatic drug
ESR: Erythrocyte sedimentation rate
FPGS: Folylpolyglutamate synthase
GI: Gastrointestinal
JDM: Juvenile dermatomyositis
JIA: Juvenile idiopathic arthritis
LC-MS/MS: Liquid chromatography–tandem mass spectrometry
MTX: Methotrexate
MTXPG: Methotrexate polyglutamate
MTXPG_1–5_: Individual methotrexate polyglutamates up to the fifth order of polyglutamation
MTXPGtotal: Total concentration of methotrexate polyglutamate
SC: Subcutaneous
